# An Architectural Battery Designed by Substituting Lithium with Second Main Group Metals (Be, Mg, Ca/Cathode) and Hybrid Oxide of Fourth Group Ones (Si, Ge, Sn/Anode) Nanomaterials Towards H_2_ Adsorption: A Computational Study

**DOI:** 10.3390/nano15130959

**Published:** 2025-06-20

**Authors:** Fatemeh Mollaamin, Majid Monajjemi

**Affiliations:** 1 Department of Biomedical Engineering, Faculty of Engineering and Architecture, Kastamonu University, Kastamonu 37150, Turkey; 2 Department of Chemical Engineering, Central Tehran Branch, Islamic Azad University, Tehran 1496969191, Iran; m_monajjemi@yahoo.com

**Keywords:** alkaline earth metal-ion battery, hydrogen adsorption, energy storage, density of states

## Abstract

Germanium/tin-containing silicon oxide [SiO–(GeO/SnO)] nanoclusters have been designed with different Si/Ge/Sn particles and characterized as electrodes for magnesium-ion batteries (MIBs) due to forming MgBe [SiO–GeO], MgBe [SiO–SnO], MgCa [SiO–GeO], and MgCa [SiO–SnO] complexes. In this work, alkaline earth metals of magnesium (Mg), beryllium (Be), and calcium (Ca) have been studied in hybrid Mg-, Be-, and Ca-ion batteries. An expanded investigation on H capture by MgBe [SiO–(GeO/SnO)] or MgCa [SiO–(GeO/SnO)] complexes was probed using computational approaches due to density state analysis of charge density differences (CDD), total density of states (TDOS), and electron localization function (ELF) for hydrogenated hybrid clusters of MgBe [SiO–GeO], MgBe [SiO–SnO], MgCa [SiO–GeO], and MgCa [SiO–SnO]. Replacing Si by Ge/Sn content can increase battery capacity through MgBe [SiO–GeO], MgBe [SiO–SnO], MgCa [SiO–GeO], and MgCa [SiO–SnO] nanoclusters for hydrogen adsorption processes and could improve the rate performances by enhancing electrical conductivity. A small portion of Mg, Be, or Ca entering the Si–Ge or Si–Sn layer to replace the alkaline earth metal sites could improve the structural stability of the electrode material at high multiplicity, thereby improving the capacity retention rate. In fact, the MgBe [SiO–GeO] remarks a small enhancement in charge transfer before and after hydrogen adsorption, confirming the good structural stability. In addition, [SiO–(GeO/SnO)] anode material could augment the capacity owing to higher surface capacitive impacts.

## 1. Introduction

These days, rechargeable multivalent-ion batteries such as magnesium-ion batteries beyond Li-ion battery technology have been attracting researchers’ attention. With a negative reduction potential of −2.37 V versus standard hydrogen electrodes and a desire for a lower dendrite formation close to that of lithium, magnesium anode can release stable performance and high energy [1,2,3]. Recently, researchers designed a quasi-solid-state magnesium-ion battery that confines the hydrogen bond network for true multivalent metal-ion storage. Mg-ion batteries offer a safe and high energy density alternative to current Li-ion batteries. Generally, aqueous batteries encounter a narrow electrochemical yield, but nonaqueous Mg-ion batteries have weak ionic conductivity [4].

Today, two Mg hybrid batteries of Mg-Li and Mg-Na have attained more attention, which merge the advantages of the high-capacitance and high-voltage cathodes of Li^+^/Na^+^ ion batteries, fast ion intercalation, and deintercalation into cathodes with high capacity [5].

It is relevant to remark that silicon hybrid inorganic compounds were examined for lithium-ion batteries [6]. Similarly, although Si-based polymer-derived ceramics were investigated in rechargeable batteries [7], there are no results related to discovering their potential for magnesium-ion batteries (MIBs).

One hopping anode material for lithium batteries is silicon, with a theoretical capacity that is more than a graphite structure [8]. But the usage of a Si anode remains moderate because of magnificent volume expansion contributing to structural deterioration [6]. The extracted polymers from ceramics, especially with a silicon backbone, might be a supreme candidate to modify the mentioned concerns [7]. Therefore, silicon oxycarbide with Si tetrahedrally coordinated to O and C was studied in rechargeable lithium-ion batteries [9,10,11,12].

Moreover, Sn-containing SiOC/Sn nanobeads are synthesized with various C/Sn elements and examined as electrodes for Mg-ion batteries [13]. In addition, the synthesis of nano hybrid materials of Sn-including Sn-SiOCN as anode materials for Mg-ion batteries was accomplished. Enhancing Sn ameliorated battery performance by reducing electrode impedance. Silicon and tin have attracted much attention in the pursuit of finding appropriate electrode materials with excellent capacity to substitute graphite in Li-ion batteries [14].

Owing to low electrical conductivity, additives such as tin (Sn) are provided to ameliorate the cycling consistency, rate performance of SiOC electrodes, and reversible capacity [15,16]. Like silicon electrodes, metallic tin electrodes endure severe volume expansion and particle association, contributing to poor cycling consistency [17]. Therefore, silicon oxycarbide ceramics during battery cycling are appropriate active matrices to buffer the volume alteration in and density of tin [18,19,20,21].

Lately, the carbide hybrid nanomaterials of Si-, Ge-, and Sn have been proposed as occupied H_2_ capture substances [22,23,24]. Whereas the polarizability of Si is more than a C atom, it is assumed that a Si–C/Si nanosurface may append to compositions more intensely in hybrids compared to the pure C nanostructures [25,26,27]. The previous investigations of energy-saving devices through H adsorption have been tailored owing to DFT calculations with a semiconductor group of Si/Ge/Sn/Pb nano-carbides [28], Mg-Al nanoalloys [29], and Al/C/Si doping of BN nanocomposites [30,31].

Si electrodes have structural deterioration and poor performance stability owing to their big volume expansion and pulverization during battery cycling that limit their usage in batteries [32,33]. So, the nanocomposites of Si-containing polymer-derived ceramics such as SiOC and SiCN have been applied instead of Si electrodes to prohibit the mentioned problems [34]. In addition, implanting tin nanomaterials in the SiOC and SiCN ceramics matrix could prevent their volume expansion during the cycling of Li-ion batteries [35,36].

In this research article, the physical and chemical attributes of the mentioned heteroclusters and hydrogenated nanoclusters of MgBe [SiO–GeO].H_2_, MgBe [SiO–SnO].H_2_, MgCa [SiO–GeO].H_2_, and MgCa [SiO–SnO].H_2_ were characterized. Regarding this context, the [SiO–GeO] or [SiO–SnO] nanocluster was modeled with hybrid alkaline earth metals of Mg/Be/Ca as the cathode materials for comparison. Then, the samples were measured to legislate their potency for the first time in Mg- batteries. Furthermore, the authors reveal how the obtained chemical viewpoints can be used to simulate other hybrid multivalent-ion batteries like MgBe- and MgCa-ion batteries.

## 2. Materials and Methods

**Figure 1:**
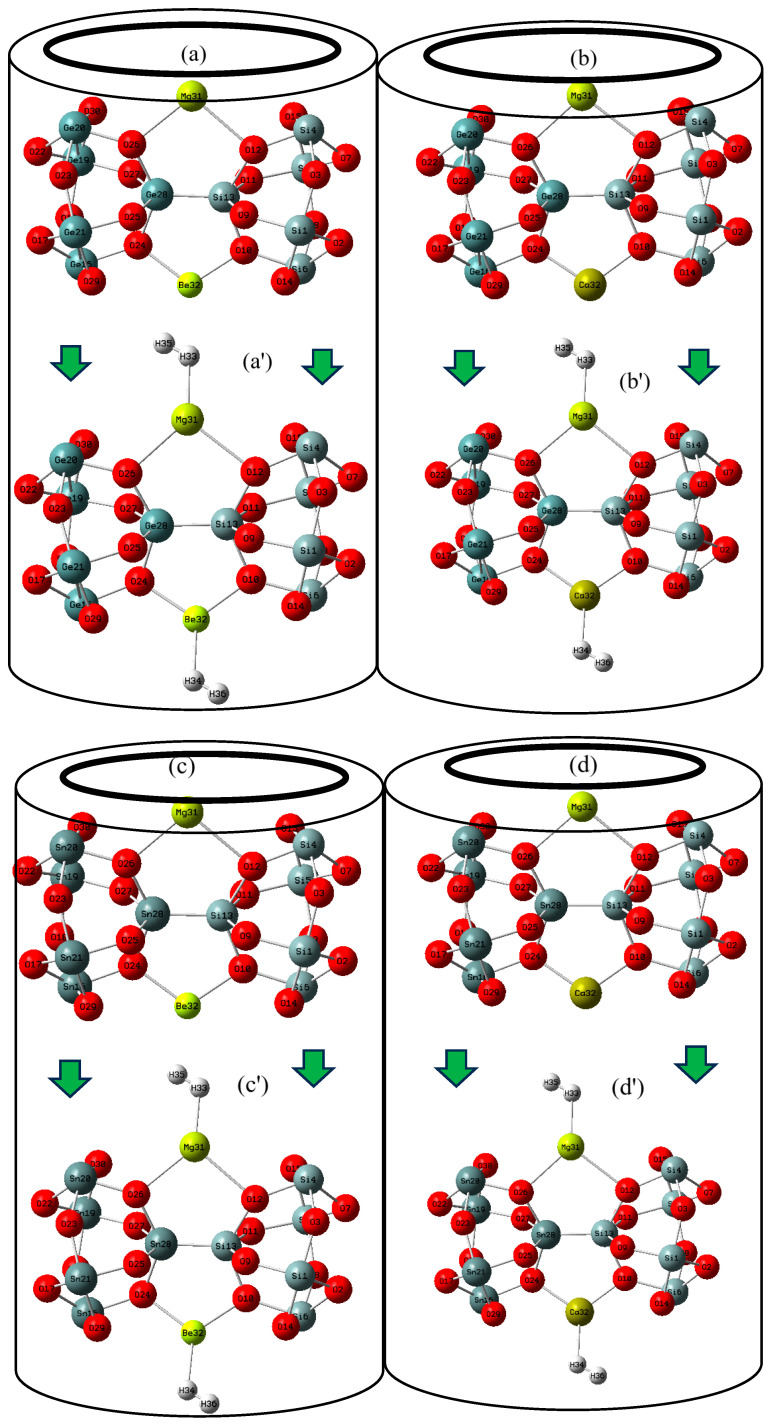
Adding Mg, Be, and Ca to [SiO–(GeO/SnO)] nanoclusters and formation of (**a**) MgBe [SiO–GeO], (**b**) MgCa [SiO–GeO], (**c**) MgBe [SiO–SnO], and (**d**) MgCa [SiO–SnO] nanoclusters towards energy storage through hydrogen adsorption as (**a′**) MgBe [SiO–SnO].H_2_, (**b′**) MgCa [SiO–GeO].H_2_, (**c′**) MgBe [SiO–SnO].H_2_, and (**d′**) MgCa [SiO–SnO].H_2_ in novel batteries.

Figure 1a–d,a′–d′ show the alkaline earth metal-based nanoclusters of MgBe [SiO–GeO], MgBe [SiO–SnO], MgCa [SiO–GeO], and MgCa [SiO–SnO], which are able to augment the hydrogen storage in battery cells or other semiconducting devices. In this investigation, the computations have been launched by the Coulomb-attenuating method–(Becke, 3-parameter, Lee–Yang–Parr) [CAM–B3LYP–D3] level of theory. Theoretical calculations have become essential tools for a comprehensive understanding of the microscopic mechanisms in energy storage materials, particularly in charge density variations and electron transport characteristic behaviors in electrode materials. In this research article, the calculations have been performed by the “CAM–B3LYP–D3” level of theory. Dispersion forces were considered under the “DFT-D3” method of Grimme with Becke–Johnson damping [37,38,39,40,41,42,43].

Figure 1a–d,a′–d′ indicate the status of H_2_ capture by MgBe [SiO–GeO], MgBe [SiO–SnO], MgCa [SiO–GeO], and MgCa [SiO–SnO] nanoclusters and hydrogen-adsorbed nanoclusters of MgBe [SiO–GeO].H_2_, MgCa [SiO–GeO].H_2_, MgBe [SiO–SnO].H_2_, and MgCa [SiO–SnO].H_2_.

The analysis of the Bader charge parameter [44] has been illustrated for the H_2_-captured hybrid clusters of MgBe [SiO–GeO].H_2_, MgBe [SiO–SnO].H_2_, MgCa [SiO–SnO].H_2_, and MgCa[SiO–SnO].H_2_ (Figure 1a–d,a′–d′) due to Gaussian 16 revision C.01 computational software [45] and GaussView 6.1 graphical program [46]. The applied basis sets for the theoretical calculations of H_2_ capture by MgBe [SiO–GeO], MgBe [SiO–SnO], MgCa [SiO–GeO], and MgCa [SiO–SnO] have been supported by LANL2DZ and 6−311+G (d,p).

One of the most significant advantages of applying (Ge/Sn)-containing SiO nanocluster as anodes/cathodes in Mg batteries is they supply the potential magnesium-ion storage in a firm [SiO–(GeO/SnO)] anode material. Mg^2+^ ions could react quickly with a Sn atom, C, and probably Si to produce various Mg-based alloys like Mg*_x_*C, Mg-Si, and Mg_2_Sn [47]. Li^+^ cation is bigger than Mg^2+^ cation, but magnesium storage mechanisms in the [SiO–(GeO/SnO)] matrix could be analogous to those of Li^+^ [47].

In this investigation, homogenously distributed germanium or tin elements can be immobilized in the [SiO–(GeO/SnO)] matrix. The Mg/Be/Ca insertion could also conclude in the cleavage of some Si–O, Ge–O, or Sn–O bonds in the [SiO–(GeO/SnO)] anode material and the expansion.

At the same time, the Mg, Be, and Ca atoms could react rapidly with a metalloid germanium or metal tin, and possibly hybrid nanocluster of [SiO–(GeO/SnO)] to form different Mg-based alloys and its hydrogenated forms of MgBe [SiO–GeO]/MgBe [SiO–GeO].H_2_ (Figure 1a,a′), MgCa [SiO–GeO]/MgCa [SiO–GeO].H_2_ (Figure 1b,b′), MgBe [SiO–SnO]/MgBe [SiO–SnO].H_2_ (Figure 1c,c′), and MgCa [SiO–SnO]/MgCa [SiO–GeO].H_2_ (Figure 1d,d′). In fact, the stable structure of the synthesized [SiO–GeO] and [SiO–GeO] hybrid materials could reduce the capacity fading of tin and germanium caused by volume increase during the charging or discharging process. Moreover, the electrical conductivity of these hybrid materials could be increased because of the high electrical conductivity of tin and germanium compared with that of silicon oxide.

## 3. Results and Discussion

### 3.1. Evaluation of Charge Density Differences (CDDs)

**Figure 2:**
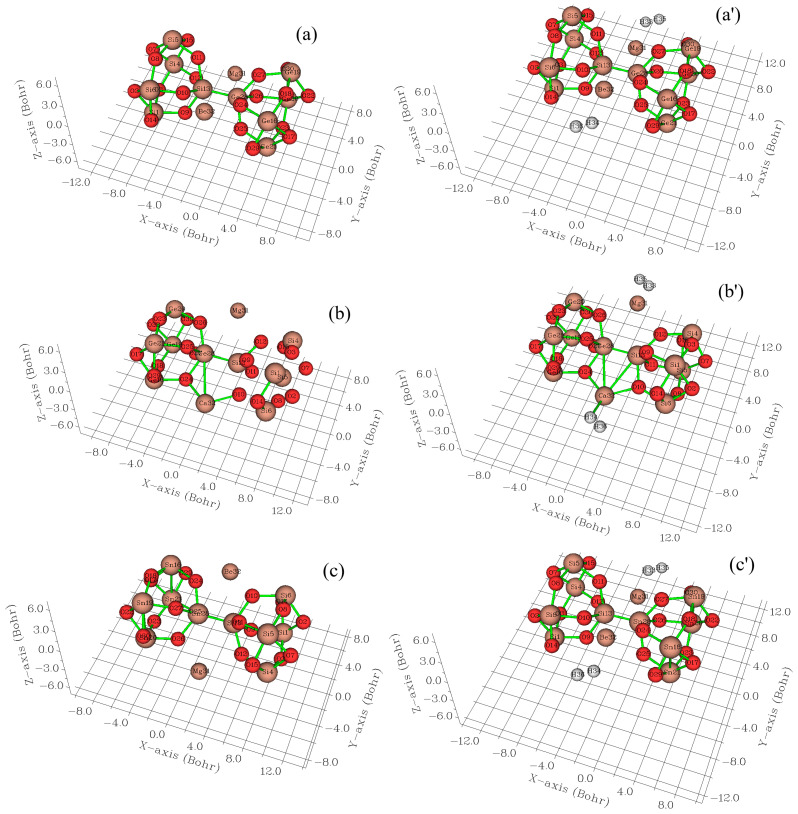

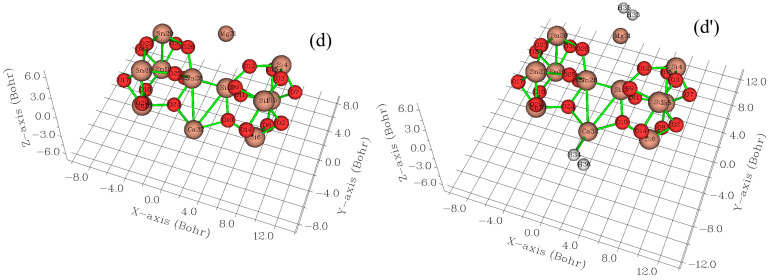
CDD graphs for (**a**) MgBe [SiO–GeO], (**a′**) MgBe [SiO–SnO].H_2_, (**b**) MgCa [SiO–GeO], (**b′**) MgCa [SiO–GeO].H_2_, (**c**) MgBe [SiO–SnO], (**c′**) MgBe [SiO–SnO].H_2_, (**d**) MgCa [SiO–SnO], and (**d′**) MgCa [SiO–SnO].H_2_ nanoclusters.

In Figure 2a–d, the CDD [48] has been displayed for MgBe [SiO–GeO], MgCa [SiO–GeO], MgBe [SiO–SnO], and MgCa [SiO–SnO] nanoclusters with the vibration in the region of about −12 to +6/+10 Bohr and for the hydrogenated forms of MgBe [SiO–GeO].H_2_, MgCa [SiO–GeO].H_2_, MgBe [SiO–SnO].H_2_, and MgCa [SiO–SnO].H_2_ (Figure 2a′–d′) in the region of about −12 to +6/+10 Bohr. Moreover, the oxygen atoms including 2, 3, 7–12, 14, 15, 17, 18, 22–27, 29, and 30 from Mg-based alloys and its hydrogenated forms of MgBe [SiO–GeO]/MgBe [SiO–GeO].H_2_, MgCa [SiO–GeO]/MgCa [SiO–GeO].H_2_, MgBe [SiO–SnO]/MgBe [SiO–SnO].H_2_, and MgCa [SiO–SnO]/MgCa [SiO–SnO].H_2_ have displayed the vibration in the region of about −12 to +6/+10 Bohr (Figure 2a–d,a′–d′).

The charge distribution has been displayed in the process of hydrogen trapping by MgBe [SiO–GeO], MgCa [SiO–GeO], MgBe [SiO–SnO], and MgCa [SiO–SnO] complexes accompanying the production of MgBe [SiO–GeO].H_2_, MgCa [SiO–GeO].H_2_, MgBe [SiO–SnO].H_2_, and MgCa [SiO–SnO].H_2_, respectively (Table 1 and Table 2). Functionalizing of Mg, Be, and Ca atoms can augment the negative atomic charge of oxygen atoms including 2,3, 7–12,14, 15, 17,18, 22–27, 29, and 30 in MgBe [SiO–GeO], MgCa [SiO–GeO], MgBe [SiO–SnO], and MgCa [SiO–SnO] nanoclusters (Table 1 and Table 2). In fact, MgBe [SiO–GeO], MgCa [SiO–GeO], MgBe [SiO–SnO], and MgCa [SiO–SnO] hybrid clusters have displayed more output than [SiO–(GeO/SnO)] [48] for electron acceptance from the electron donor of hydrogen atoms of 33–36 (Table 1 and Table 2).

MgBe [SiO–GeO] and MgCa [SiO–GeO] have shown a Bader charge of −1.545 and −1.733 coulomb before hydrogen adsorption and −1.548 and −1.688 coulomb after H_2_ capture. In addition, the fluctuation in the charge density values for MgBe [SiO–SnO] and MgCa [SiO–SnO] has shown a Bader charge of −1.677 and −1.712 coulomb before hydrogen adsorption and −1.661 and −1.662 coulomb after hydrogen adsorption. The differences in the charge density value for these hybrid clusters are estimated as follows: ΔQ_MgBe [SiO–GeO]_ = −0.003, ΔQ_MgCa [SiO–GeO]_ = +0.045, ΔQ_MgBe [SiO–SnO]_ = +0.016, and ΔQ_MgCa [SiO–SnO]_ = +0.05 coulomb. Therefore, the MgBe [SiO–GeO] complex could have the most gravity for electron receiving owing to H_2_ capture. In fact, the MgBe [SiO–GeO] remarks a small enhancement in charge transfer before and after hydrogen adsorption, confirming the good structural stability.

### 3.2. Total Density of States

The notion of “density of states (DOS) and original total density of states (TDOS)” of an isolated system (a molecule) can be written as follows [49]:

(1) $$TDOS\;\left(E\right)=\sum_{i}\delta \;(E-\epsilon_{i\;})$$

The normalized Gaussian function is defined as follows:

(2) $$G\left(x\right)=\frac{1}{c\sqrt{2\pi }}e^{-\frac{x^{2}}{{2c}^{2}}}\;\mathrm{where}\;c=\frac{FWHM}{2\sqrt{2lnx}}$$

“FWHM (full width at half maximum)” is an adjustable parameter in “Multiwfn” [50,51]. Furthermore, the curve maps of “broadened partial DOS (PDOS)” and “overlap DOS (OPDOS)” are valuable for visualizing orbital composition analysis, the “PDOS function of fragment *A*” is defined as follows:

(3) $${PDOS}_{A}\;\left(E\right)=\sum_{i}\Xi_{i,A}\;F\;(E-\epsilon_{i\;})$$

where “$\Xi_{i,A}$ is the composition of fragment *A* in orbital *i*”. The “OPDOS between fragment *A* and *B*” is defined as follows:

(4) $${OPDOS}_{A,B}\;\left(E\right)=\sum_{i}X_{A,B}^{i}\;F\;(E-\epsilon_{i})$$

where “$X_{A,B}^{i}$ is the composition of total cross term between fragment *A* and *B* in orbital *i*”.

**Figure 3:**
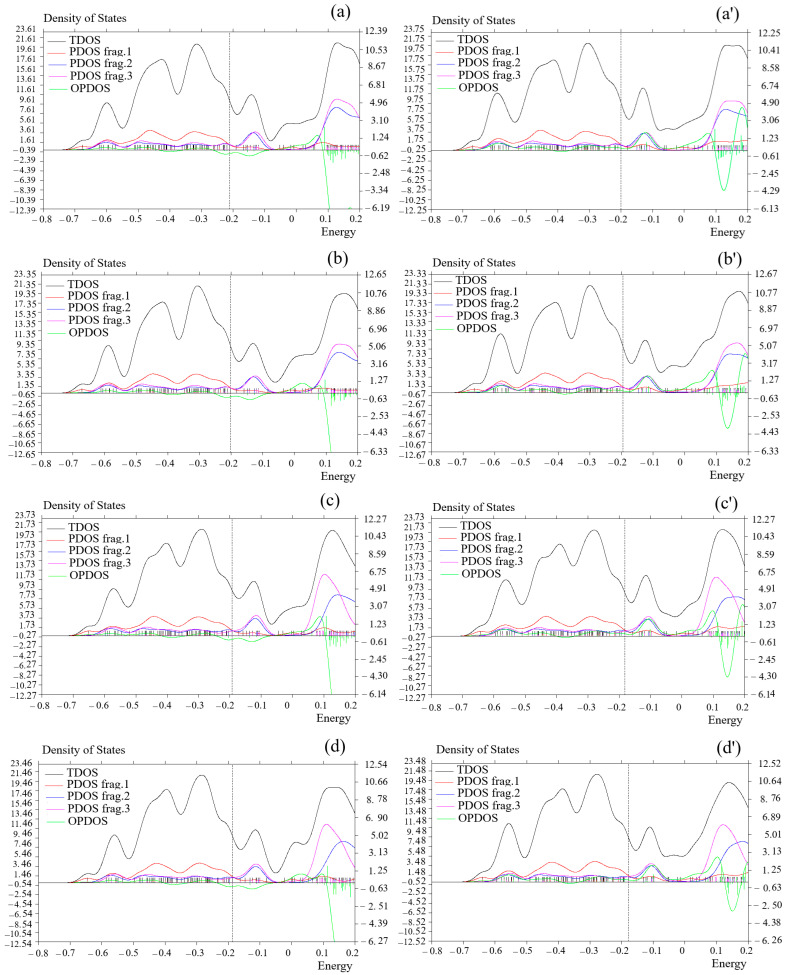
OPDOS/PDOS/TDOS graphs of (**a**) MgBe [SiO–GeO], (**a′**) MgBe [SiO–SnO].H_2_, (**b**) MgCa [SiO–GeO], (**b′**) MgCa [SiO–GeO].H_2_, (**c**) MgBe [SiO–SnO], (**c′**) MgBe [SiO–SnO].H_2_, (**d**) MgCa [SiO–SnO], and (**d′**) MgCa [SiO–SnO].H_2_ nanoclusters.

Considering hydrogen trapping by MgBe [SiO–GeO], MgCa [SiO–GeO], MgBe [SiO–SnO], and MgCa [SiO–SnO] nanoclusters, the total density of states was evaluated. This factor can demonstrate the existence of significant chemical interactions often on the “convex side” (Figure 3a–d,a′–d′).

MgBe [SiO–GeO], MgCa [SiO–GeO], MgBe [SiO–SnO], and MgCa [SiO–SnO] (Figure 3a–d) and hydrogenated nanoclusters containing MgBe [SiO–GeO].H_2_, MgCa [SiO–GeO].H_2_, MgBe [SiO–SnO].H_2_, and MgCa [SiO–SnO].H_2_ (Figure 3a′–d′) have shown the steepest maximum TDOS surrounding −0.30, −0.40, and −0.60 a.u. owing to the covalent bond between Mg/Be and Mg/Ca with a [SiO–(GeO/SnO)] nanocluster with maximum density of states of ≈22.

Frag.1 was displayed for O(9)–O(12), Si(13), O(24)–O(27) and Ge(28), and Mg(31)/X(32) (Y = Be, Ca) in Figure 3a–d and H(36) to H(36) in Figure 3a′–d′. Frag.2 exhibited the fluctuation in Si(1) and Si(4)–Si(6) beside the analogous elements of Frag.1 in Figure 3a–d,a′–d′. In addition, the fluctuation in Ge(16)/Sn(16), Ge(19)/Sn(19) to Ge(21)/Sn(21), O(17), O(18), O(22), O(23), O(29), and O(30) is considered in Figure 3a–d,a′–d′ through Frag.3.

### 3.3. Electron Localization Function Analysis

Nevertheless, the distinction between deduced/raised electron delocalization/localization into cyclic π-conjugated sets stays encouraging for ELF [52]. The larger the electron localization is in an area, the more likely the electron movement is restricted within it. Therefore, they could be discerned from the ones away if electrons are totally centralized [53,54,55].

**Figure 4:**
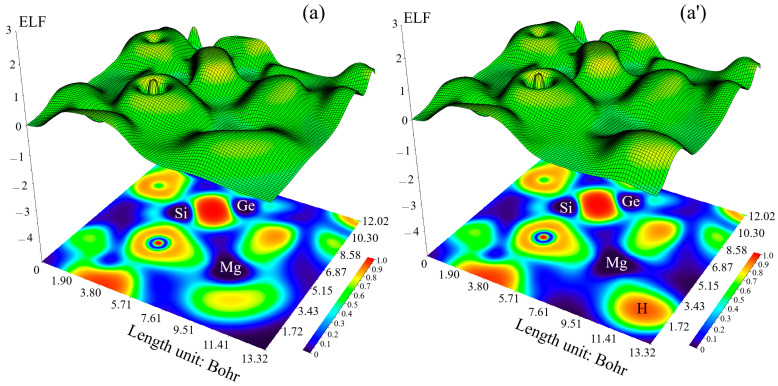

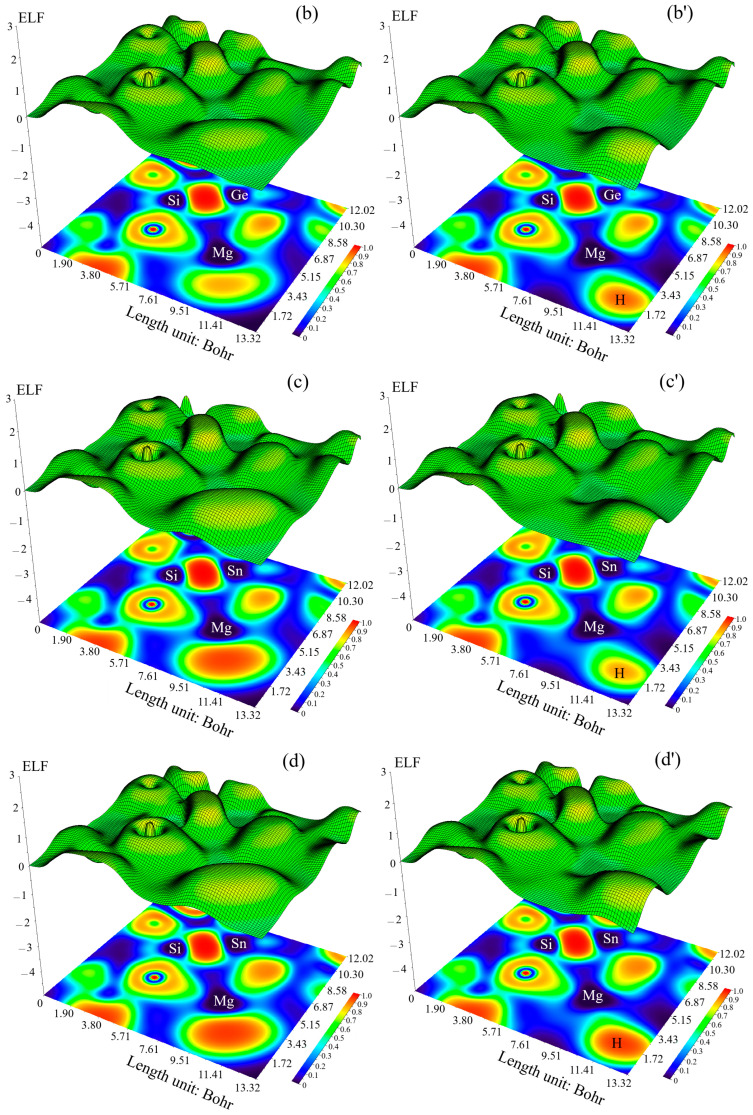
The shaded map of ELF graphs for (**a**) MgBe [SiO–GeO], (**a′**) MgBe [SiO–SnO].H_2_, (**b**) MgCa [SiO–GeO], (**b′**) MgCa [SiO–GeO].H_2_, (**c**) MgBe [SiO–SnO], (**c′**) MgBe [SiO–SnO].H_2_, (**d**) MgCa [SiO–SnO], and (**d′**) MgCa [SiO–SnO].H_2_ nanoclusters.

Trapping of hydrogens by MgBe [SiO–GeO], MgCa [SiO–GeO], MgBe [SiO–SnO], and MgCa [SiO–SnO] complexes (Figure 4a–d) towards the formation of MgBe [SiO–GeO].H_2_, MgCa [SiO–GeO].H_2_, MgBe [SiO–SnO].H_2_, and MgCa [SiO–SnO].H_2_ could be described by ELF graphs using Multiwfn [50,51] due to achieving their delocalization/localization characterizations [52] of electrons and chemical bonds (Figure 4a′–d′).

MgBe [SiO–GeO] (Figure 4a), MgBe [SiO–GeO].H_2_ (Figure 4a′), MgCa [SiO–GeO] (Figure 4b), MgCa [SiO–GeO].H_2_ (Figure 4b′), MgBe [SiO–SnO] (Figure 4c), MgBe [SiO–SnO].H_2_ (Figure 4c′), MgCa [SiO–SnO] (Figure 4d), and MgCa [SiO–SnO].H_2_ (Figure 4d′) have demonstrated electron delocalization through an isosurface map with labeling elements of O(10), O(12), Si(13), O(24), O(26), Ge(28) or Sn(28), X(31)(X = Mg), Y(32) (Y = Be or Mg) and H(33), H(34), H(35), and H(36). In fact, the counter map of ELF can approve that MgBe [SiO–GeO], MgCa [SiO–GeO], MgBe [SiO–SnO], and MgCa [SiO–SnO] nanoclusters may augment the efficiency during H_2_ capture towards the formation of MgBe [SiO–GeO].H_2_, MgCa [SiO–GeO].H_2_, MgBe [SiO–SnO].H_2_, and MgCa [SiO–SnO].H_2_ (Table 1 and Table 2).

The observed map in Figure 4 corresponds to the formation of X_2_Y (X = Mg, Be, Ca; Y = Si, Ge, Sn) based on the electrochemical reaction:

(5) $$2X^{2+}+Y+4e^{-}\to X_{2}Y$$

The existence of Ge or Sn in the [SiO–GeO] or [SiO–SnO] matrix may decrease the electrode polarization and improve the battery performance [56].

In addition, the intermolecular orbital overlap integral is significant in the illustration of intermolecular charge transfer, which can compute “HOMO–HOMO” and “LUMO–LUMO” overlap integrals between the H_2_ molecules and heterostructures of MgBe [SiO–GeO], MgBe [SiO–GeO].H_2_, MgCa [SiO–GeO], MgCa [SiO–GeO].H_2_, MgBe [SiO–SnO], MgBe [SiO–SnO].H_2_, MgCa [SiO–SnO], and MgCa [SiO–SnO].H_2_ nanoclusters. The layered germanium/tin–silicon oxide improved by the alkaline earth metals of magnesium, beryllium, and calcium has indicated the structural stability of Mg-, Be-, and Ca-ion batteries through the reported stability energy in Table 3. A small portion of Mg, Be, or Ca entering the Si–Ge or Si–Sn layer to replace the alkaline earth metal sites could improve the structural stability of the electrode material at high multiplicity, thereby improving the capacity retention rate of 645.7713, 543.9274, 505.4256, and 440.8573 mAhg^−1^ for MgBe [SiO–GeO], MgCa [SiO–GeO], MgBe [SiO–SnO], and MgCa [SiO–SnO] complexes, respectively (Table 3).

Furthermore, the binding energies of Mg-, Be-, and Ca-ions and [SiO–GeO] or [SiO–SnO] heteroclusters have been calculated, which are large enough to prohibit metal atom clustering (Table 3). The charge surfaces produced at the metal spot, which can insert a dipole in the H2 molecule, can attach to the H_2_ molecule by the ion quadrupole and by ion-induced dipole interactions. The consequences display that MgBe [SiO–GeO], MgCa [SiO–GeO], MgBe [SiO–SnO], and MgCa [SiO–SnO] complexes can save the H_2_ molecule. These investigations could introduce a viewpoint of modeling new 3D hydrogen-saving materials with a [SiO–GeO] or [SiO–SnO] cluster doped with alkaline earth metals as the structure units.

## 4. Conclusions

H_2_ trapping by the hybrid materials of MgBe [SiO–GeO], MgCa [SiO–GeO], MgBe [SiO–SnO], and MgCa [SiO–SnO] was studied by computational approaches. The changes in the charge density defined a substantial charge transfer in MgBe [SiO–GeO], MgCa [SiO–GeO], MgBe [SiO–SnO], and MgCa [SiO–SnO]. Enhancing Mg, Be, or Ca to cell batteries could augment energy saving in cell storage. In addition, H bond accepting sites by MgBe [SiO–GeO], MgCa [SiO–GeO], MgBe [SiO–SnO], and MgCa [SiO–SnO] can alleviate parasitic H evolution in H_2_O electrolytes in Mg-, Be-, and Ca-ion batteries. The results of this research article represent that the architectural design of XY(GeSiO) (X = Mg/Y = Be,Ca) can augment the capacity of battery cells. A small portion of Mg, Be, or Ca entering the Si–Ge or Si–Sn layer to replace the alkaline earth metal sites could improve the structural stability of the electrode material at high multiplicity, thereby improving the capacity retention rate of 645.7713, 543.9274, 505.4256, and 440.8573 mAhg^−1^ for MgBe [SiO–GeO], MgCa [SiO–GeO], MgBe [SiO–SnO], and MgCa [SiO–SnO] complexes, respectively. So, the MgBe [SiO–GeO] remarks a small enhancement in charge transfer before and after hydrogen adsorption, confirming the good structural stability. This research article is useful for designing and constructing Mg hybrid batteries with high power density/energy density with excellent cycle stability and will represent a perspective for the industrial application of Mg hybrid batteries.

## Figures and Tables

**Table 1:** The atomic charge (Q/coulomb) for MgBe [SiO–GeO], MgBe [SiO–GeO].H_2_, MgCa [SiO–GeO], and MgCa [SiO–GeO].H_2_ nanoclusters.

MgBe [SiO–GeO]		MgBe [SiO–GeO].H_2_		MgCa [SiO–GeO]		MgCa [SiO–GeO].H_2_	
Atom	Q	Atom	Q	Atom	Q	Atom	Q
Si1	1.4393	Si1	1.4305	Si1	1.4372	Si1	1.4325
O2	−0.6703	O2	−0.6828	O2	−0.6788	O2	−0.6881
O3	−0.8343	O3	−0.8327	O3	−0.8340	O3	−0.8329
Si4	1.3740	Si4	1.3631	Si4	1.366	Si4	1.3546
Si5	1.4332	Si5	1.4236	Si5	1.4286	Si5	1.4205
Si6	1.4172	Si6	1.4043	Si6	1.3943	Si6	1.3921
O7	−0.6767	O7	−0.6788	O7	−0.6763	O7	−0.6795
O8	−0.8532	O8	−0.8514	O8	−0.8475	O8	−0.8450
O9	−0.8153	O9	−0.8220	O9	−0.8197	O9	−0.8221
O10	−0.9649	O10	−0.9656	O10	−1.2365	O10	−1.2353
O11	−0.8329	O11	−0.8361	O11	−0.8281	O11	−0.8270
O12	−1.0175	O12	−1.0178	O12	−1.0194	O12	−1.0193
Si13	1.5445	Si13	1.5476	Si13	1.5089	Si13	1.5121
O14	−0.7512	O14	−0.7688	O14	−0.7804	O14	−0.7884
O15	−0.7733	O15	−0.7962	O15	−0.7745	O15	−0.7959
Ge16	1.3562	Ge16	1.3394	Ge16	1.3435	Ge16	1.3411
O17	−0.6284	O17	−0.6676	O17	−0.6324	O17	−0.6341
O18	−0.7884	O18	−0.7866	O18	−0.7822	O18	−0.7821
Ge19	1.3576	Ge19	1.3384	Ge19	1.3549	Ge19	1.3444
Ge20	1.3304	Ge20	1.3158	Ge20	1.3243	Ge20	1.3063
Ge21	1.3682	Ge21	1.3580	Ge21	1.3704	Ge21	1.3623
O22	−0.6836	O22	−0.6495	O22	−0.6884	O22	−0.6937
O23	−0.7965	O23	−0.7968	O23	−0.7970	O23	−0.7977
O24	−0.9093	O24	−0.9021	O24	−1.1820	O24	−1.1798
O25	−0.8050	O25	−0.8164	O25	−0.8135	O25	−0.8152
O26	−0.9793	O26	−0.9859	O26	−0.9811	O26	−0.9853
O27	−0.8325	O27	−0.8270	O27	−0.8143	O27	−0.8169
Ge28	1.2053	Ge28	1.2023	Ge28	1.1979	Ge28	1.1995
O29	−0.7461	O29	−0.7516	O29	−0.7978	O29	−0.8025
O30	−0.7284	O30	−0.7719	O30	−0.7262	O30	−0.7438
Mg31	1.2824	Mg31	1.2834	Mg31	1.2514	Mg31	1.2686
Be32	0.9790	Be32	0.9635	Ca32	1.7324	Ca32	1.6878
		H33	−0.0845			H33	−0.0824
		H34	−0.0343			H34	−0.1340
		H35	0.1936			H35	0.1901
		H36	0.1630			H36	0.1893

**Table 2:** The atomic charge (Q/coulomb) for MgBe [SiO–SnO], MgBe [SiO–SnO].H_2_, MgCa [SiO–SnO], and MgCa [SiO–SnO].H_2_ nanoclusters.

MgBe [SiO–SnO]		MgBe [SiO–SnO].H_2_		MgCa [SiO–SnO]		MgCa [SiO–SnO].H_2_	
Atom	Q	Atom	Q	Atom	Q	Atom	Q
Si1	1.4285	Si1	1.4183	Si1	1.4300	Si1	1.4257
O2	−0.6719	O2	−0.6897	O2	−0.6895	O2	−0.6988
O3	−0.8351	O3	−0.8318	O3	−0.8320	O3	−0.8304
Si4	1.3636	Si4	1.3479	Si4	1.3597	Si4	1.3429
Si5	1.4168	Si5	1.4086	Si5	1.4167	Si5	1.4093
Si6	1.4029	Si6	1.3881	Si6	1.3827	Si6	1.3819
O7	−0.6886	O7	−0.6904	O7	−0.6830	O7	−0.6892
O8	−0.8536	O8	−0.8503	O8	−0.8450	O8	−0.8419
O9	−0.8227	O9	−0.8313	O9	−0.8304	O9	−0.8334
O10	−0.9564	O10	−0.9576	O10	−1.2393	O10	−1.2369
O11	−0.8390	O11	−0.8425	O11	−0.8324	O11	−0.8316
O12	−1.0083	O12	−1.0051	O12	−1.0091	O12	−1.0063
Si13	1.3627	Si13	1.3705	Si13	1.3426	Si13	1.3483
O14	−0.7749	O14	−0.7901	O14	−0.8001	O14	−0.8099
O15	−0.7646	O15	−0.8013	O15	−0.7757	O15	−0.8049
Sn16	1.6457	Sn16	1.6109	Sn16	1.6092	Sn16	1.6015
O17	−0.8062	O17	−0.8108	O17	−0.7944	O17	−0.7967
O18	−0.8870	O18	−0.8859	O18	−0.8719	O18	−0.8711
Sn19	1.6753	Sn19	1.6503	Sn19	1.6533	Sn19	1.6358
Sn20	1.6269	Sn20	1.5864	Sn20	1.5903	Sn20	1.5527
Sn21	1.6774	Sn21	1.6537	Sn21	1.6238	Sn21	1.6110
O22	−0.8309	O22	−0.8272	O22	−0.8107	O22	−0.8102
O23	−0.8919	O23	−0.8907	O23	−0.8810	O23	−0.8797
O24	−0.9885	O24	−0.9824	O24	−1.2527	O24	−1.2501
O25	−0.9185	O25	−0.9202	O25	−0.9201	O25	−0.9219
O26	−1.0596	O26	−1.0603	O26	−1.0678	O26	−1.0690
O27	−0.9570	O27	−0.9598	O27	−0.9403	O27	−0.9417
Sn28	1.6716	Sn28	1.6607	Sn28	1.6348	Sn28	1.6351
O29	−0.8873	O29	−0.9011	O29	−0.9249	O29	−0.9273
O30	−0.8926	O30	−0.9072	O30	−0.8887	O30	−0.9027
Mg31	1.1567	Mg31	1.2116	Mg31	1.1344	Mg31	1.1928
Be32	0.9065	Be32	0.9048	Ca32	1.7117	Ca32	1.6622
		H33	−0.0736			H33	−0.0707
		H34	−0.0386			H34	−0.1403
		H35	0.1767			H35	0.1734
		H36	0.1597			H36	0.1924

**Table 3:** Stability energy (E_s_, kcal/mol), binding energy (E_b_, kcal/mol), dipole moment (D,debye), E_LUMO_ (eV), E_HOMO_ (eV), and energy gap (∆E = E_LUMO_ − E_HOMO_) (eV) and cell capacity (C, mAh g^−1^) for MgBe [SiO–GeO], MgBe [SiO–SnO].H_2_, MgCa [SiO–GeO], MgCa [SiO–GeO].H_2_, MgBe [SiO–SnO], MgBe [SiO–SnO].H_2_, MgCa [SiO–SnO], and MgCa [SiO–SnO].H_2_ heteroclusters.

Heteroclusters	E_s_ × 10^−3^	E_b_ × 10^−3^	D	E_HOMO_	E_LUMO_	∆E	C
MgBe [SiO–GeO]	−976.9153	−1.4649	3.4528	−5.7388	−5.1779	0.5609	645.7713
MgBe [SiO–GeO].H_2_	−978.3802		3.7950	−5.4447	−4.8919	0.5528	
MgCa [SiO–GeO]	−990.5851	−1.4469	0.9860	−5.5242	−4.9432	0.5809	543.9274
MgCa [SiO–GeO].H_2_	−992.0320		2.2704	−5.3185	−4.7421	0.5764	
MgBe [SiO–SnO]	−975.2794	−1.4631	7.4382	−5.2431	−4.6503	0.5927	505.4256
MgBe [SiO–SnO].H_2_	−976.7425		7.4859	−4.9463	−4.3148	0.6315	
MgCa [SiO–SnO]	−988.9556	−1.4453	6.3492	−5.0786	−4.4365	0.6420	440.8573
MgCa [SiO–SnO].H_2_	−990.4009		6.1184	−4.8506	−4.1993	0.6513	

## Data Availability

Data are contained within the article.
